# Supplementary material for: Content-rich biological network constructed by mining PubMed abstracts
Source: BMC Bioinformatics. 2004 Oct 8;5:147. doi: 10.1186/1471-2105-5-147 (PMC528731; doi:10.1186/1471-2105-5-147)
Supplement: Additional File 2 — The original results of the above study (non-essential files are deleted to keep the file size under the limit set by BMC bioinformatics). [file 1471-2105-5-147-S2.bz2 › chilibotAdditionalFile2/dip30/257ID8242751E1020/html/CCND1_CDKN1A.html]

 


 **CCND1** and **CDKN1A** 
  
Found 633 abstracts in PubMed, retrieved 30.  
 

 What does Google say? 
 PDF only 
| .edu only 

---

**Interactive relationship** (e.g. stimulation, inhibition, etc)

**Inhibitory relationship**- Semi quantitative reverse transcription polymerase chain reaction RT PCR analysis revealed that 20 epi 5 OH 2 D 3 induced the expression of retinoic acid receptor beta and p21 Cip1  [ **CDKN1A** ] , and down regulated the expression of cyclin D1  [ **CCND1** ]  resulting in decreased phosphorylation of retinoblastoma protein pRB.  Ref: 12787874 Biochem Pharmacol, 2003
- Moreover, Rb phosphorylation, which lies immediately downstream of the cyclin D1  [ **CCND1** ]  Cdk4 p21WAF1 Cip1  [ **CDKN1A** ]  complex, was blocked either by Sp1 overexpression or antisense p21WAF1 Cip1  [ **CDKN1A** ] .  Ref: 12796485 J Biol Chem< MedlineTA>J Biol Chem, 2003
**Stimulatory relationship**- In p53 null or p21 WAF1  [ **CDKN1A** ]  null cells marked apoptosis occurred after SN38 alone, and was additionally enhanced by Bcl  [ **CCND1** ]  xl knockdown in p21 WAF1  [ **CDKN1A** ]  null cells but not in p53 null cells.  Ref: 12855666 Clin Cancer Res, 2003
- The bypass of TNF induced G1 arrest seems to be related to the increase in the stability of cyclin D  [ **CCND1** ]  bound CDK complexes, increasing the total amount of CDK2 4 and 6 complexes and leading to a functional down titration of the p21 waf1  [ **CDKN1A** ]  molecules.  Ref: 12761882 J Cell Biochem, 2003
**Neutral relationship**

**Non-interactive relationship** (e.g. studied together, co-existance, homology, etc.)

- In our study 86 tonsil cancers were analysed for HPV status by sequence analysis of polymerase chain reaction products and for the expression of cell cycle proteins p53, p21 CIP1  [ **CDKN1A** ]  WAF1  [ **CDKN1A** ] , pRb, p16 INK4A, cyclin D1  [ **CCND1** ]  and p27 KIP1 by immunohistochemistry.  Ref: 12845651 Int J Cancer, 2003
- These events were accompanied by down regulation of the Raf 1 MEK ERK pathway as well as diminished expression of Bcr Abl and cyclin D1  [ **CCND1** ] , cleavage of p21 CIP1  [ **CDKN1A** ]  and pRb, and induction of the stress related kinases JNK and p38 MAPK.  Ref: 12893773 BloodBlood, 2003
- The down regulation of Bcl  [ **CCND1** ]  2 and or the upregulation of p53 and p21 WAF 1  [ **CDKN1A** ]  are certainly one of the important modes of apoptosis induction by taxanes.  Ref: 12769774 Curr Med Chem Anti Canc Agents< MedlineTA>Curr Med Chem Anti Canc Agents, 2003
- Abnormalities in G1 S transition in cell cultures have been attributed to alterations in ErbB erythroblastic leukaemia viral v erb b oncogene homologue, avian signalling, cyclin D1  [ **CCND1** ]  overexpression or disturbance of the p21 WAF1  [ **CDKN1A** ]  p21 mediated cell cycle arrest induced by p53.  Ref: 12825853 Breast Cancer Res Treat, 2003
- SN38 induced p53, Bax, Bcl  [ **CCND1** ]  xl, and p53 dependent p21 WAF1  [ **CDKN1A** ]  protein accumulation.  Ref: 12855666 Clin Cancer Res, 2003
- Additionally, the proteins of the p53 regulated genes, p21 WAF1  [ **CDKN1A** ]  and Bax, were increased with a similar time, while Bcl  [ **CCND1** ]  2 and Bcl  [ **CCND1** ]  X L expression was lowered.  Ref: 12753950 Biol Cell, 2003
- Changes in the expression of caspase 3, bax, bcl  [ **CCND1** ]  2 and p21cip waf  [ **CDKN1A** ]  were assessed by quantitative Western blotting.  Ref: 12888913 Int J Oncol, 2003
- These data identify c Myc, cyclin D1  [ **CCND1** ] , p21 WAF1  [ **CDKN1A** ]  CIP1  [ **CDKN1A** ]  and cyclin E Cdk2 as central components of estrogen regulation of cell cycle progression and hence as potential downstream targets that contribute to the role of estrogen in oncogenesis.  Ref: 12790780 Endocr Relat Cancer< MedlineTA>Endocr Relat Cancer, 2003
- Sp1 blocked assembly of cyclin D1  [ **CCND1** ]  Cdk4 p21WAF1 Cip1  [ **CDKN1A** ]  complex formation whose integrity is critical for G1 > ; S transition.  Ref: 12796485 J Biol Chem< MedlineTA>J Biol Chem, 2003
- Most thymomas and thymic carcinomas were negative for Bcl  [ **CCND1** ]  XL, p21WAF, 1 CIP1  [ **CDKN1A** ]  and cyclin D1  [ **CCND1** ] .  Ref: 12877732 Histopathology, 2003
- Cyclin D1  [ **CCND1** ]  up regulation and p21 WAF1  [ **CDKN1A** ]  CIP1  [ **CDKN1A** ]  down regulation occur early in UC related carcinogenesis.  Ref: 12827612 Hum Pathol, 2003
- Experimental Design Using wild type HCT116, p53 null, Bax null, or p21 WAF1  [ **CDKN1A** ]  null isogenic derivatives, we measured expression of regulators of cellular response, and associated growth arrests or apoptosis, after SN38 treatment, with or without antisense mediated Bcl  [ **CCND1** ]  xl knockdown.  Ref: 12855666 Clin Cancer Res, 2003
- The molecular pathways of butyrate and related next generation synthetic SCFAs in mediating these effects have not been fully elucidated, but HDAC inhibition is associated with regulation of critical cell cycle regulators, such as cyclin D  [ **CCND1** ] , p21 CIP1  [ **CDKN1A** ]  WAF1  [ **CDKN1A** ] , and p27 KIP1.  Ref: 12769690 Curr Cancer Drug Targets, 2003
- AIMS To correlate the expression of a series of apoptotic and oncogene markers including p53, Bcl  [ **CCND1** ]  2, BAX, Bcl  [ **CCND1** ]  XL, p21WAF, 1 CIP1  [ **CDKN1A** ] , cyclin D1  [ **CCND1** ] , HER 2 neu in thymic epithelial tumours with histological type, stage and resectability and to determine whether the information on HER 2 neu would be valuable in identifying patients who are eligible for anti HER 2 neu treatment.  Ref: 12877732 Histopathology, 2003
- Sp1 Inhibits proliferation and induces apoptosis in vascular smooth muscle cells by repressing p21WAF1 Cip1  [ **CDKN1A** ]  transcription and cyclin D1  [ **CCND1** ]  Cdk4 p21WAF1 Cip1  [ **CDKN1A** ]  complex formation.  Ref: 12796485 J Biol Chem< MedlineTA>J Biol Chem, 2003
- In addition, the latter induces changes in gene expression by up regulating p53, Waf 1  [ **CDKN1A** ] , Cas, Dap kinase and caspases and 14 and down regulating Bcl  [ **CCND1** ]  2.  Ref: 12887065 Biol Chem, 2003
- On the other hand, prolonged ERK activity was observed with a subsequent increase of cyclin D1  [ **CCND1** ]  and p21 Cip  [ **CDKN1A** ]  WAF1  [ **CDKN1A** ]  upon treating with 100 micro M of ZnCl2 in MSSM, and this was associated with non proliferation.  Ref: 12816758 Am J Physiol Gastrointest Liver Physiol< MedlineTA>Am J Physiol Gastrointest Liver Physiol, 2003
- The combination effectively up regulated caspase 3 and bax and down regulated bcl  [ **CCND1** ]  2 and p21cip waf  [ **CDKN1A** ] .  Ref: 12888913 Int J Oncol, 2003
- We have shown that abolition of p21 WAF1  [ **CDKN1A** ]  mediated drug induced senescence or antisense mediated Bcl  [ **CCND1** ]  xl knockdown can both, independently, enhance the apoptotic response of colorectal cancer cells to SN38 in vitro.  Ref: 12855666 Clin Cancer Res, 2003
- p XSC and p XSeSG were significantly more effective in the upregulation of pro apoptotic genes, such as p21 CIP1  [ **CDKN1A** ]  WAF1  [ **CDKN1A** ] , p27 KIP1, APO 1, and Caspase 3, while downregulating cell growth regulatory genes, such as c myc, cyclin D1  [ **CCND1** ] , cyclin D2 and proliferating cell nuclear antigen PCNA.  Ref: 12844480 Carcinogenesis< MedlineTA>Carcinogenesis, 2003
- In these conditions the TNF induced increase of p21 waf1  [ **CDKN1A** ]  is not sufficient to inhibit the high amount of cyclin D  [ **CCND1** ]  bound complexes.  Ref: 12761882 J Cell Biochem, 2003
- It is unclear whether and how cyclin D1  [ **CCND1** ]  and or p21 WAF1  [ **CDKN1A** ]  CIP1  [ **CDKN1A** ]  dysregulation contribute to ulcerative colitis UC related inflammation and colorectal carcinogenesis.  Ref: 12827612 Hum Pathol, 2003
- QUC showed cyclin D1  [ **CCND1** ]  negative weak staining and p21 WAF1  [ **CDKN1A** ]  CIP1  [ **CDKN1A** ]  surface epithelial and upper third crypt staining expression similar to that of normal colorectum.  Ref: 12827612 Hum Pathol, 2003
- These results suggest that the suppression of cell cycle progression in the G1 phase by 5GG was mediated in MCF 7 cells, at least in part, by either the inhibition of cyclin D  [ **CCND1** ]  CDK4 and cyclin E CDK2 activity or the induction of the CDK inhibitors p27 Kip and p21 Cip  [ **CDKN1A** ] .  Ref: 12781329 Biochem Pharmacol, 2003
- These events culminated in activation of the caspase cascade, manifested by poly ADP ribose polymerase, p21 CIP1  [ **CDKN1A** ]  WAF1  [ **CDKN1A** ] , p27 KIP, Bcl  [ **CCND1** ]  2, and retinoblastoma protein degradation.  Ref: 12839953 Cancer Res, 2003
- The differential regulations of cell growth, ERK activities, and cyclin D1  [ **CCND1** ]  and p21 Cip  [ **CDKN1A** ]  WAF1  [ **CDKN1A** ]  inductions were also observed in serum enriched medium containing higher zinc concentrations.  Ref: 12816758 Am J Physiol Gastrointest Liver Physiol< MedlineTA>Am J Physiol Gastrointest Liver Physiol, 2003
- Moreover, this ERK activation and cyclin D1  [ **CCND1** ]  and p21 Cip  [ **CDKN1A** ]  WAF1  [ **CDKN1A** ]  induction were abolished by PD98059 pre treatment.  Ref: 12816758 Am J Physiol Gastrointest Liver Physiol< MedlineTA>Am J Physiol Gastrointest Liver Physiol, 2003
- n = 11 were studied with cyclin D1  [ **CCND1** ]  and p21 WAF1  [ **CDKN1A** ]  CIP1  [ **CDKN1A** ]  immunohistochemistry.  Ref: 12827612 Hum Pathol, 2003
- Also, t BuOOH treatment caused an elevation in the levels of the pro apoptotic proteins p53 and p21 WAF 1  [ **CDKN1A** ]  and a reduction in the levels of the anti apoptotic protein bcl  [ **CCND1** ]  2 compared to their levels in control HCN1 A cells, while pretreatment with nicotinamide reduced p53 and p21 WAF 1  [ **CDKN1A** ]  levels even in the presence of t BuOOH.  Ref: 12782109 Neurotoxicology< MedlineTA>Neurotoxicology, 2003
- In conclusion, AUC shows up regulated cyclin D1  [ **CCND1** ]  and p21 WAF1  [ **CDKN1A** ]  CIP1  [ **CDKN1A** ]  expression.  Ref: 12827612 Hum Pathol, 2003
